# Elucidating Host–Pathogen Interactions Based on Post-Translational Modifications Using Proteomics Approaches

**DOI:** 10.3389/fmicb.2015.01312

**Published:** 2015-11-20

**Authors:** Vaishnavi Ravikumar, Carsten Jers, Ivan Mijakovic

**Affiliations:** ^1^Systems and Synthetic Biology Division, Department of Biology and Biological Engineering, Chalmers University of Technology, Gothenburg, Sweden; ^2^Department of Systems Biology, Technical University of Denmark, Lyngby, Denmark; ^3^Novo Nordisk Foundation Center for Biosustainability, Technical University of Denmark, Hørsholm, Denmark

**Keywords:** co-cultures, pathogenesis, bacteria, proteomics, post-translational modifications

## Abstract

Microbes with the capability to survive in the host tissue and efficiently subvert its innate immune responses can cause various health hazards. There is an inherent need to understand microbial infection patterns and mechanisms in order to develop efficient therapeutics. Microbial pathogens display host specificity through a complex network of molecular interactions that aid their survival and propagation. Co-infection states further lead to complications by increasing the microbial burden and risk factors. Quantitative proteomics based approaches and post-translational modification analysis can be efficiently applied to gain an insight into the molecular mechanisms involved. The measurement of the proteome and post-translationally modified proteome dynamics using mass spectrometry, results in a wide array of information, such as significant changes in protein expression, protein abundance, the modification status, the site occupancy level, interactors, functional significance of key players, potential drug targets, etc. This mini review discusses the potential of proteomics to investigate the involvement of post-translational modifications in bacterial pathogenesis and host–pathogen interactions.

## Introduction

Phylogenetically diverse microorganisms such as bacteria, viruses or protozoans commonly pose as “pathogens.” They have the capacity to survive in the host tissue, adapt to and suppress the host innate immune responses, thus constantly challenging human health and welfare. The normal microbial flora that inhabits specific regions in the host organism is essential for the proper functioning of the host. In contrast, pathogens elicit specific unnatural responses in the host system. Over the years, the field of infection biology has thus generated much interest, especially with the current emergence of various multi-drug resistant strains. The infection cycle of a pathogen comprises mainly of three stages, namely introduction of the pathogen into the host cell or tissue, establishment of the pathogen within the host and dissemination within the host. Despite continuous improvements in the techniques developed to identify and elucidate infection patterns, there is a pressing demand for more efficient advancements to interpret the molecular cues during infection. Mass spectrometry based proteomics, through the years, has been instrumental in deciphering the molecular mechanisms underlying host–pathogen interactions (Yang et al., 2015). Development of high-resolution instruments and improvements in experimental techniques has helped expand the scope of infection biology.

The delicate balance that exists between the host immune responses and the pathogen can be tipped toward the latter by the co-existence of multiple microbial species capable of colonizing the same host niche. Bacteria have been identified in a wide range of symbiotic associations such as pathogenic, mutualistic or as commensals. The state of “natural selection” enforces interspecies specific interactions that differ significantly from the behavior of the individual species. Bacterial co-cultures or mixed populations find numerous benefits (1) they are commonly employed in the biotechnology industry such as in waste water treatment (Tijhuis et al., 1994), biogas plants (Klocke et al., 2007), production of vitamin C (Ma et al., 2014); (2) they form an important part of the human gut microbiota (Putignani et al., 2014). Adversely, symbiotic interactions can also be unfavorable, resulting in host invasion and damage (Chowdhury et al., 2010; Kluge et al., 2012). Thus systematic analysis of bacteria in co-culture conditions is essential to obtain an understanding of microbial behavior under conditions of infection. Routine strategies employed by pathogens to defeat host immune responses generally include adherence to host cell surfaces, followed by invasion and subsequent production and secretion of enterotoxins along with exhibiting host molecular mimicry mechanisms (Zhang et al., 2005). Protein secretion systems are thus pivotal in infection (Tseng et al., 2009). Targeting any one of these systems could help curb transmission of the disease or elimination of the pathogen. The following sections in this review describe the implications of various post-translational modifications (PTMs) on bacterial virulence and the proteomics technologies available to investigate such events along with the associated technical challenges.

## Post-Translational Modifications

Protein PTMs are known to play diverse roles in the cellular milieu. Bacterial virulence is orchestrated by a multitude of PTMs. Some of the most common PTMs critical for the infection process are reviewed here.

### Phosphorylation

Phosphorylation is a well-characterized and ubiquitous PTM that is crucial for most functions occurring in a bacterial cell. Apart from regulating metabolic pathways, phosphorylation has also been seen to be involved in various virulence mechanisms. The existence of Hanks-type Ser/Thr kinases in bacteria has generated a huge interest in the field of infection biology. Pathogenic strains such as *Streptococcus* spp., *Pseudomonas aeruginosa*, *Mycobacteria*, *Yersinia* spp., to name a few, employ Ser/Thr kinase-mediated host–pathogen interactions to mediate diverse cellular networks required for adhesion to and invasion of the host. While the exact mechanism of infection is not yet well understood, the mode of infection has been speculated to follow three basic modes: (1) phosphorylation of host proteins, (2) disruption of host defense mechanisms due to kinase activity, and lastly (3) essential role of Ser/Thr kinases by unrealized processes (Canova and Molle, 2014). The global phosphoproteome of a number of pathogenic bacteria such as *Corynebacterium glutamicum* (Bendt et al., 2003), *Campylobacter jejuni* (Voisin et al., 2007), *Klebsiella pneumoniae* (Lin et al., 2009), *Mycobacterium tuberculosis* (Prisic et al., 2010), *Streptomyces coelicolor* (Parker et al., 2010), to name a few, have been analyzed. Changes that occur in the host phosphoproteome upon bacterial infection have also been investigated (Schmutz et al., 2013; Scholz et al., 2015). Dynamics of the bacterial phosphoproteome at the time of infection and post infection would be an interesting avenue to embark upon.

Secretion systems play a vital role during pathogenesis in a large number of bacteria. *Yersinia enterocolitica* and other species encode for a protein kinase A, YopO, which is secreted into the host via the type III secretion system. This kinase helps resist phagocytosis by macrophages via disruption of host cytoskeletal elements (Juris et al., 2000; Grosdent et al., 2002). This kinase was also reported to phosphorylate actin and otubain, resulting in inhibition of phagocytosis (Juris et al., 2006). Mutation of the kinase domain has been shown to reduce lethality during infection (Galyov et al., 1993; Wiley et al., 2006). Similarly, Stk1 from *Staphylococcus aureus* has been shown to phosphorylate numerous host substrates involved in cell cycle signaling or apoptotic pathways (Miller et al., 2010). SteC of *Salmonella enterica* serovar Typhimurium, like YopO, induces reorganization of actin filaments in the host on infection (Odendall et al., 2012). Host immune cell responses depend on the proper functioning of the NF-κB pathway. *Legionella pneumophila* LegK acts as an inflammatory agent and interferes with the NF-κB pathway (Ge et al., 2009). Likewise, protein kinases NleH1 and NleH2 from enteropathogenic *Escherichia coli* work by inhibiting the transcription factor, NF-κB (Royan et al., 2010). Phosphorylation of the central core of type II fatty acid synthase (FASH) in *Mycobacterium tuberculosis*, catalyzed by the kinase KasB, governs the physiopathology of tuberculosis (Vilcheze et al., 2014). Apart from phosphorylation by Ser/Thr Hanks-type kinases, bacterial two-component systems involving phosphorylation on histidine-aspartate residues (TCS) form a major adaptive mechanism in pathogenic strains. For example, CovRS (the control of virulence regulator/sensor kinase) in the human pathogen group A *Streptococcus* is fundamental for virulence (Horstmann et al., 2015). Interestingly, cysteine protein phosphorylation events are also reported to mediate bacterial virulence. The SarA/MarA staphylococcal accessary regulator A, part of the family of global transcriptional regulators (MgrA), is phosphorylated/dephosphorylated by the *Staphylococcus aureus* kinase/phosphatase pair Stk1-Stp1 and speculated to play a crucial role in shifting the intracellular redox balance, contributing to virulence (Sun et al., 2012).

Cognate to kinase activity is the activity of phosphatases, making phosphorylation a reversible and tightly regulated PTM. In many organisms, protein phosphatases act as essential virulence determinants, thus playing a central role in infection and dissemination. YopH tyrosine phosphatase from *Yersinia* is involved in the dephosphorylation of the focal adhesion complexes and essential for antiphagocytosis (Persson et al., 1999). SptP tyrosine phosphatase of *Salmonella typhimurium* was observed to be required for virulence in murine models (Kaniga et al., 1996). The phosphothreonine lyase protein, OspF, from *Shigella flexneri* irreversibly phosphorylates members of the MAPK and ERK pathway, subsequently affecting the innate immune system (Reiterer et al., 2011). Dephosphorylation of tyrosine kinases such as the BY-kinase Wzc-ca from *E. coli* K12 by Wzb causes increased capsular polysaccharide formation which can further act as a poor immunogen (Whitmore and Lamont, 2012; Hansen et al., 2013).

### Acylation

Acetylation can be used as a mechanism to modulate phosphorylation-based signaling. *Yersinia* species use a serine/threonine acetyltransferase, YopJ, to interfere with host MAPK kinase signaling by acetylating serine and threonine residues in the activation loop thereby preventing phosphorylation-dependent activation (Mukherjee et al., 2006). YopJ homologs are widely distributed in both mammal and plant pathogens suggesting that inhibition of host kinases by serine/threonine acetylation could be a common strategy for bacterial pathogens (Lewis et al., 2011). Lysine acetylation and succinylation have in recent years been shown to be abundant modifications in bacteria, and there are some indications that they could be important for bacterial pathogenesis. In *E. coli*, the transcription factor RcsB that controls colanic acid capsule synthesis, is acetylated on lysine thereby reducing its DNA binding activity (Thao et al., 2010). A global study indicated that lysine acetylation is involved in regulation of cell wall fatty acids synthesis in *M. tuberculosis*, which in turn is implicated in pathogenicity (Liu et al., 2014). In addition, a lysine deacetylase (MRA_1161) mutant exhibits a defect in biofilm formation (Liu et al., 2014). KasA, a protein involved in biofilm formation, is modified by another type of acylation namely lysine succinylation. Further, a number of proteins involved in antibiotic resistance are succinylated (Xie et al., 2015). With only few functional studies, it is still unclear to what extent lysine acetylation and succinylation contribute to bacterial virulence.

### Ubiquitination

Ubiquitination is an important PTM in Eukarya and regulates several processes including key cell defense systems. Ubiquitin is a small polypeptide (78 amino acids) that can be covalently linked to primarily lysine. Ubiquitination requires the activities of an E1 activating enzyme, an E2 conjugating enzyme and an E3 ligase (Ashida et al., 2014). Ubiquitin contains seven lysines and can itself be ubiquitinated leading to formation of polyubiquitin chains with various linkages that in turn dictates biological function (Ashida et al., 2014). Bacterial pathogens have developed several ways of targeting the host ubiquitin system including the use of Eukaryotic-like and novel E3 ligases as well as de-ubiquitinating enzymes. The *S. flexneri* E3 ligase IpaH9.8 reduces the NF-κB-mediated inflammatory response by polyubiquitination of NEMO, a protein involved in NF-κB activation, thereby targeting it for degradation (Ashida et al., 2010). Additionally, IpaH9.8 appears to modulate gene expression via ubiquitination of the splicing factor U2AF^35^ (Okuda et al., 2005; Seyedarabi et al., 2010). In *S. enterica* infection, ubiquitinated protein aggregates are formed near the *Salmonella*-containing vacuole which targets it for autophagic degradation. This is countered by SseL, a deubiquitinase, that deubiquitinates the protein aggregates (Mesquita et al., 2012). The pathogens can also use the host ubiquitination system to modify their own proteins. To control the timing of effector protein-mediated functions, *S. enterica* SopE, that targets RHO-GTPases leading to membrane ruffling, is ubiquitinated and degraded earlier than the protein SptP that prevents membrane ruffling after invasion (Kubori and Galan, 2003). Another *S. enterica* effector protein, the phosphatase SopB, can be mono-ubiquitinated at six positions and this in turn modulates its cellular location and enzyme activity (Knodler et al., 2009).

### AMPylation

AMPylation is the covalent attachment of AMP to a threonine or tyrosine residue. *Vibrio parahaemolyticus* effector VopS uses ATP to covalently modify Rho-GTPases with AMP on threonine. This is turn affects its interaction with downstream signaling proteins leading to inhibition of actin assembly (Yarbrough et al., 2009). To exploit host cell vesicle transport, *L. pneumophila* SidM activates the small GTPase Rab1 by AMPylation on a tyrosine, and when Rab1 is no longer needed, it is de-AMPylated by SidD, and subsequently targeted for degradation by polyubiquitination (Neunuebel et al., 2011). Host GTPases are also targeted by another novel, reversible PTM, namely phosphocholination. *L. pneumophila* effectors AnkX and Lpg0696 phosphocholinate and de-phosphocholinate Rab1 and Rab35, respectively (Mukherjee et al., 2011; Tan et al., 2011).

### Alkylation

Protein alkylation is the addition of alkyl groups on specific amino acids, notably methyl on arginine and lysine (methylation) and the lipids farnesyl or geranylgeranyl isoprenyl on cysteine (prenylation). Histone proteins are regulated by lysine methylation, and this is also a target for bacterial pathogens. The *Bacillus anthracis* protein BaSET trimethylates histone H1 on lysine and thereby reduces activation of NF-κB response elements (Mujtaba et al., 2013). Prenylation confers hydrophobicity to its substrate proteins and target them to membranes. This is employed by *L. pneumophila* to assure correct cellular localization of its effector AnkB. Host-mediated farnesylation of AnkB, targets it to the cytosolic face of the *Legionella*-containing vacuole and this in turn is essential for its function (Price et al., 2010).

### Eliminylation

*Shigella flexneri* effector protein OspF, a phosphothreonine lyase, interferes with host signaling by irreversibly dephosphorylating MAP kinases. In this PTM termed eliminylation, not only the phosphate but also the hydroxyl group of threonine is removed, thereby preventing any future phosphorylation. It irreversibly inactivates the kinase (Li et al., 2007).

### Glycosylation

Protein glycosylation is now a well-established modification in bacteria. O-linked glycosylation of flagellar proteins has been linked to virulence in pathogenic bacteria. In the opportunistic pathogen *Burkholderia cenocepacia*, glycosylation of the flagellar protein FliC reduces binding to the host TLR5 receptor, thereby weakening the immune response. Additionally, a non-glycosylated FliC mutant exhibits a defect in biofilm formation (Hanuszkiewicz et al., 2014). *Helicobacter pylori* flagellar proteins are also glycosylated, and this is essential for virulence (Schirm et al., 2003). In a systematic study of *H. pylori* glycoproteins, 26 proteins with diverse roles in pathogenesis were identified, arguably indicating a broader array of mechanisms for glycosylation-based virulence (Champasa et al., 2013).

## Potential of Proteomics

Assessment of host–pathogen interactions is beneficial from a surveillance and diagnostic perspective. Pursuing the proteomic signatures of pathogenic bacteria during infection states could reveal dynamic changes that occur in the proteome or PTM proteome, which are often induced due to the selection pressure occurring during the interaction with the host. High-resolution mass spectrometry based proteomics has the ability to bridge the gap between genomics and the physiological mechanisms of behavior (Dove, 1999). Global and quantitative analysis of the pathogen proteome provides an in depth understanding of the molecular events that are differentially regulated during the onset of infection. Dynamic changes occurring at the proteome level, quantitative post-translational modification analysis are some of the basic and widely used applications of the shotgun proteomics technology. The high-throughput “omics” technology also allows for identification of macromolecular components important for specific cell-to-cell communications (Chait, 2006), for monitoring metabolic shifts and revealing metabolic pathways involved and/or activated during specific stages of infection. The conventional shot-gun proteomics or the bottom-up approach involves protein extraction from the organism of interest followed by digestion with a suitable endoprotease and subsequent analysis by LC-MS/MS. Entire bacterial proteomes and factors that play a key role in certain virulence pathways can be identified and analyzed conveniently by employing this approach (Tracz et al., 2013; Alvarez Hayes et al., 2015). Conversely, top-down approaches that involve the analysis of intact proteins have also been adopted to characterize bacterial proteomes (Ansong et al., 2013). Additionally, while challenging, top-down proteomics can be employed to study PTMs such as phosphorylation, acetylation, etc, occurring in the bacterial proteome as well as the host proteome upon infection (Kelleher, 2004; Zhang and Ge, 2011).

Microbial proteomics additionally aids in detection of biomarkers which could possibly act as targets for drug based therapy (Guest et al., 2013). Proteomics studies conducted on the effects of drug dosage are also crucial for acquiring information about the real-time *in vivo* scenario, thus assisting in the development of antimicrobial drugs. Targeted proteomics is also a powerful method for focusing on a specific subset of proteins involved in infection and resistance mechanisms, allowing for mechanistic questions to be answered.

## Current Technology

A limited number of non-proteomics based methodologies are currently employed for the investigation of population dynamics of binary mixed cultures (Kluge et al., 2012), namely, Fluorescence *In Situ* Hybridization (FISH; Rogers et al., 2000), Real-Time PCR (Higuchi et al., 1993), Flow Cytometry (Müller et al., 1995) and Terminal Restriction Fragment Length Polymorphism (T-RFLP; Schmidt et al., 2007). Metaproteome analysis of mixed cultures using 2-DE followed by LC-ESI-MS has been utilized to identify diverse microbial communities in the environment (Benndorf et al., 2007; Kluge et al., 2012). High-resolution mass spectrometry based approaches are routinely employed to study bacterial infection models or bacterial co-cultures. Global subcellular protein profiling has been applied to reveal subcellular distributions of proteins, subsequently used to reconstruct functional networks and to identify new targets responsible for pathogenicity (Mawuenyega et al., 2005). *De novo* sequencing has been applied for peptide/protein identification when the DNA sequence coverage of a particular organism has been unavailable or incomplete (Wilmes and Bond, 2004). Centrifugation followed by detergent solubilization (Fernandez-Arenas et al., 2007); ImmunoMagnetic Separation (IMS) using anti-IgG-coated Dynabeads^TM^ in combination with antisera (Twine et al., 2006); or Fluorescence-Activated Cell Sorting (FACS; Becker et al., 2006) based approaches are routinely used for separation of bacterial cells from host cells. Pulse-chase experiments using ^35^S-labeled methionine or cysteine can also be employed to monitor changes occurring at the level of protein synthesis or protein turn-over (Schmidt and Volker, 2011). Quantification of proteome dynamics post infection can be done by label free approaches [for example, Luo et al. (2014) identified 2125 phosphopeptides and quantified 253 and 344 up-regulated phosphorylation events from PRRSV-infected pulmonary alveolar macrophages 12 and 36 h postinoculation respectively by employing label free approaches (Luo et al., 2014)] or by other label based relative quantitation methods such as iTRAQ (isobaric tagging for relative and absolute quantitation) or SILAC (stable isotope labeling with amino acids in cell culture; Shui et al., 2009). In the paper by Shui et al. (2009), a total of 1286 proteins were identified from murine macrophages during *M. tuberculosis* infection, of which 463 were identified by both SILAC and iTRAQ labeling strategies. Targeted proteomics, that forms a part of the newer generation proteomics approaches, can also be applied to study specific changes occurring in the host or bacterial cell by applying multiple reaction monitoring mass spectrometry (MRM-MS). Lange et al. (2008) employ multiple reaction monitoring to understand the dynamics of virulence factors from the Gram-positive bacterium *Streptococcus pyogenes*. Similarly, Karlsson et al. (2012) use selected reaction monitoring mass spectrometry (SRM-MS) to decipher the *S. pyogenes* proteome. Mass-spectrometry based imaging techniques form a fresh avenue of approach for studying differences in microbial communities (Wilmes and Bond, 2009).

## Technical Challenges

In the field of infection biology, the characterization of microbes existing as interacting communities is extremely challenging but essential (Kluge et al., 2012). Genome is a static entity, while the proteome is dynamic, creating a higher order of complexity in whole proteome analysis in comparison to the genome. The presence of PTMs further increases the functional diversity of an organism’s proteome. Furthermore, only a fraction of a given organism’s proteome is modified at any given time point, making it harder to detect or quantify certain events. Limitations with respect to instrumentation can also decrease the mass range of detection, hence losing out on critical biological information. Another major limiting factor is the absence of a complete genome sequence for many relevant bacterial species in complex environmental samples. As a consequence, limitations in the analysis of protein modifications could occur mainly due to the partial sequence coverage. When investigating bacterial proteomes under conditions of infection, extra care must be taken to ensure that the ratio of abundance of the bacterial to eukaryal proteins is not disproportional, as the bacterial proteome can easily be drowned in the noise of the highly abundant eukaryal proteins. Furthermore, the complete recoverability of the low number of bacterial cells under such conditions is difficult. This is a challenge for the PTM analysis, as generally a high amount of protein is required for enrichment of modified peptides. Additionally, mammalian-based infection studies are limited by ethical constraints, and a delicate balance must be struck to gain mechanistic understanding in a true *in vivo* setup.

## Perspectives

Developments in experimental methodologies and instrumentation have made it possible to efficiently investigate host–pathogen interactions. However, there is still a necessity for further advances in instrumentation, technology and improvements in data processivity to be able to (a) obtain a complete or sufficiently high coverage of the pathogen proteome starting with low input material; (b) conveniently identify and differentiate between organism-specific proteomes to be able to monitor the species-specific changes occurring; and (c) routinely monitor alterations occurring at the post-translation level to reveal the pathways relevant for drug targeting. Proteomics based mass-spectrometry is a powerful tool that helps obtain a functional understanding of microbial co-operativity, interaction, evolution, physiological changes and cellular functioning. Quantitative proteomic profiling of host–pathogen interactions and discovery mode proteomics, in combination with bioinformatics, have been shown as an effective approach to illuminate key factors intrinsic to host invasion and disease propagation. Additionally, data-dependent analysis or targeted approaches show a high degree of promise and are now being applied for biomarker discovery and clinical applications. The potential for definite target selection and validation should be further exploited to reveal basic solutions to important biological issues. Furthermore, proteomics analysis of PTMs from the bacterial perspective would be an interesting avenue to venture into. Apart from a few global phosphoproteome analyses, currently there are not many reports on other post-translationally modified pathogenic bacterial proteomes. Resolving some of the above-described technical challenges could aid in unraveling physiological pathways affected by PTMs that help in virulence.

### Conflict of Interest Statement

The authors declare that the research was conducted in the absence of any commercial or financial relationships that could be construed as a potential conflict of interest.

## References

[B1] Alvarez Hayes J. Lamberti Y. Surmann K. Schmidt F. Volker U. Rodriguez M. E. (2015). Shotgun proteome analysis of *Bordetella pertussis* reveals a distinct influence of iron availability on the bacterial metabolism, virulence, and defense response. Proteomics 15, 2258–2266. 10.1002/pmic.201400512 25755163

[B2] Ansong C. Wu S. Meng D. Liu X. Brewer H. M. Deatherage Kaiser B. L. (2013). Top-down proteomics reveals a unique protein S-thiolation switch in *Salmonella typhimurium* in response to infection-like conditions. Proc. Natl. Acad. Sci. U.S.A. 110, 10153–10158. 10.1073/pnas.1221210110 23720318 PMC3690903

[B3] Ashida H. Kim M. Sasakawa C. (2014). Exploitation of the host ubiquitin system by human bacterial pathogens. Nat. Rev. Microbiol. 12, 399–413. 10.1038/nrmicro3259 24801936

[B4] Ashida H. Kim M. Schmidt-Supprian M. Ma A. Ogawa M. Sasakawa C. (2010). A bacterial E3 ubiquitin ligase IpaH9.8 targets NEMO/IKKgamma to dampen the host NF-kappaB-mediated inflammatory response. Nat. Cell Biol. 12, 66–73; sup 61–69. 10.1038/ncb2006 20010814 PMC3107189

[B5] Becker D. Selbach M. Rollenhagen C. Ballmaier M. Meyer T. F. Mann M. (2006). Robust Salmonella metabolism limits possibilities for new antimicrobials. Nature 440, 303–307. 10.1038/nature04616 16541065

[B6] Bendt A. K. Burkovski A. Schaffer S. Bott M. Farwick M. Hermann T. (2003). Towards a phosphoproteome map of *Corynebacterium glutamicum*. Proteomics 3, 1637–1646. 10.1002/pmic.200300494 12923788

[B7] Benndorf D. Balcke G. U. Harms H. von Bergen M. (2007). Functional metaproteome analysis of protein extracts from contaminated soil and groundwater. ISME J. 1, 224–234. 10.1038/ismej.2007.39 18043633

[B8] Canova M. J. Molle V. (2014). Bacterial serine/threonine protein kinases in host–pathogen interactions. J. Biol. Chem. 289, 9473–9479. 10.1074/jbc.R113.529917 24554701 PMC3974997

[B9] Chait B. T. (2006). Chemistry. Mass spectrometry: bottom-up or top-down? Science 314, 65–66. 10.1126/science.1133987 17023639

[B10] Champasa K. Longwell S. A. Eldridge A. M. Stemmler E. A. Dube D. H. (2013). Targeted identification of glycosylated proteins in the gastric pathogen *Helicobacter pylori* (Hp). Mol. Cell. Proteomics 12, 2568–2586. 10.1074/mcp.M113.029561 23754784 PMC3769331

[B11] Chowdhury F. Begum Y. A. Alam M. M. Khan A. I. Ahmed T. Bhuiyan M. S. (2010). Concomitant enterotoxigenic *Escherichia coli* infection induces increased immune responses to *Vibrio cholerae* O1 antigens in patients with cholera in Bangladesh. Infect. Immun. 78, 2117–2124. 10.1128/IAI.01426-09 20176796 PMC2863502

[B12] Dove A. (1999). Proteomics: translating genomics into products? Nat. Biotechnol. 17, 233–236. 10.1038/6972 10096288

[B13] Fernandez-Arenas E. Cabezon V. Bermejo C. Arroyo J. Nombela C. Diez-Orejas R. (2007). Integrated proteomics and genomics strategies bring new insight into *Candida albicans* response upon macrophage interaction. Mol. Cell. Proteomics 6, 460–478. 10.1074/mcp.M600210-MCP200 17164403

[B14] Galyov E. E. Hakansson S. Forsberg A. Wolf-Watz H. (1993). A secreted protein kinase of *Yersinia pseudotuberculosis* is an indispensable virulence determinant. Nature 361, 730–732. 10.1038/361730a0 8441468

[B15] Ge J. Xu H. Li T. Zhou Y. Zhang Z. Li S. (2009). A Legionella type IV effector activates the NF-kappaB pathway by phosphorylating the IkappaB family of inhibitors. Proc. Natl. Acad. Sci. U.S.A. 106, 13725–13730. 10.1073/pnas.0907200106 19666608 PMC2728961

[B16] Grosdent N. Maridonneau-Parini I. Sory M. P. Cornelis G. R. (2002). Role of Yops and adhesins in resistance of *Yersinia enterocolitica* to phagocytosis. Infect. Immun. 70, 4165–4176. 10.1128/IAI.70.8.4165-4176.2002 12117925 PMC128122

[B17] Guest P. C. Gottschalk M. G. Bahn S. (2013). Proteomics: improving biomarker translation to modern medicine? Genome Med. 5, 17. 10.1186/gm421 23445684 PMC3706758

[B18] Hansen A. M. Chaerkady R. Sharma J. Diaz-Mejia J. J. Tyagi N. Renuse S. (2013). The *Escherichia coli* phosphotyrosine proteome relates to core pathways and virulence. PLoS Pathog. 9:e1003403. 10.1371/journal.ppat.1003403 23785281 PMC3681748

[B19] Hanuszkiewicz A. Pittock P. Humphries F. Moll H. Rosales A. R. Molinaro A. (2014). Identification of the flagellin glycosylation system in *Burkholderia cenocepacia* and the contribution of glycosylated flagellin to evasion of human innate immune responses. J. Biol. Chem. 289, 19231–19244. 10.1074/jbc.M114.562603 24841205 PMC4081957

[B20] Higuchi R. Fockler C. Dollinger G. Watson R. (1993). Kinetic PCR analysis: real-time monitoring of DNA amplification reactions. Biotechnology (N Y) 11, 1026–1030. 7764001 10.1038/nbt0993-1026

[B21] Horstmann N. Sahasrabhojane P. Saldana M. Ajami N. J. Flores A. R. Sumby P. (2015). Characterization of the effect of the histidine kinase CovS on response regulator phosphorylation in group A *Streptococcus*. Infect. Immun. 83, 1068–1077. 10.1128/IAI.02659-14 25561708 PMC4333468

[B22] Juris S. J. Rudolph A. E. Huddler D. Orth K. Dixon J. E. (2000). A distinctive role for the *Yersinia protein* kinase: actin binding, kinase activation, and cytoskeleton disruption. Proc. Natl. Acad. Sci. U.S.A. 97, 9431–9436. 10.1073/pnas.170281997 10920208 PMC16881

[B23] Juris S. J. Shah K. Shokat K. Dixon J. E. Vacratsis P. O. (2006). Identification of otubain 1 as a novel substrate for the *Yersinia protein* kinase using chemical genetics and mass spectrometry. FEBS Lett. 580, 179–183. 10.1016/j.febslet.2005.11.071 16364312

[B24] Kaniga K. Uralil J. Bliska J. B. Galan J. E. (1996). A secreted protein tyrosine phosphatase with modular effector domains in the bacterial pathogen *Salmonella typhimurium*. Mol. Microbiol. 21, 633–641. 8866485 10.1111/j.1365-2958.1996.tb02571.x

[B25] Karlsson C. Malmstrom L. Aebersold R. Malmstrom J. (2012). Proteome-wide selected reaction monitoring assays for the human pathogen *Streptococcus pyogenes*. Nat. Commun. 3, 1301. 10.1038/ncomms2297 23250431 PMC3535367

[B26] Kelleher N. L. (2004). Top-down proteomics. Anal. Chem. 76, 197A–203A. 10.1021/ac0415657 15190879

[B27] Klocke M. Mahnert P. Mundt K. Souidi K. Linke B. (2007). Microbial community analysis of a biogas-producing completely stirred tank reactor fed continuously with fodder beet silage as mono-substrate. Syst. Appl. Microbiol. 30, 139–151. 10.1016/j.syapm.2006.03.007 16697135

[B28] Kluge S. Hoffmann M. Benndorf D. Rapp E. Reichl U. (2012). Proteomic tracking and analysis of a bacterial mixed culture. Proteomics 12, 1893–1901. 10.1002/pmic.201100362 22623171

[B29] Knodler L. A. Winfree S. Drecktrah D. Ireland R. Steele-Mortimer O. (2009). Ubiquitination of the bacterial inositol phosphatase, SopB, regulates its biological activity at the plasma membrane. Cell. Microbiol. 11, 1652–1670. 10.1111/j.1462-5822.2009.01356.x 19614667 PMC2762020

[B30] Kubori T. Galan J. E. (2003). Temporal regulation of salmonella virulence effector function by proteasome-dependent protein degradation. Cell 115, 333–342. 10.1016/S0092-8674(03)00849-3 14636560

[B31] Lange V. Malmstrom J. A. Didion J. King N. L. Johansson B. P. Schafer J. (2008). Targeted quantitative analysis of *Streptococcus pyogenes* virulence factors by multiple reaction monitoring. Mol. Cell. Proteomics 7, 1489–1500. 10.1074/mcp.M800032-MCP200 18408245 PMC2494906

[B32] Lewis J. D. Lee A. Ma W. Zhou H. Guttman D. S. Desveaux D. (2011). The YopJ superfamily in plant-associated bacteria. Mol. Plant Pathol. 12, 928–937. 10.1111/j.1364-3703.2011.00719.x 21726386 PMC6640427

[B33] Li H. Xu H. Zhou Y. Zhang J. Long C. Li S. (2007). The phosphothreonine lyase activity of a bacterial type III effector family. Science 315, 1000–1003. 10.1126/science.1138960 17303758

[B34] Lin M. H. Hsu T. L. Lin S. Y. Pan Y. J. Jan J. T. Wang J. T. (2009). Phosphoproteomics of *Klebsiella pneumoniae* NTUH-K2044 reveals a tight link between tyrosine phosphorylation and virulence. Mol. Cell. Proteomics 8, 2613–2623. 10.1074/mcp.M900276-MCP200 19696081 PMC2816022

[B35] Liu F. Yang M. Wang X. Yang S. Gu J. Zhou J. (2014). Acetylome analysis reveals diverse functions of lysine acetylation in *Mycobacterium tuberculosis*. Mol. Cell Proteomics 13, 3352–3366. 10.1074/mcp.M114.041962 25180227 PMC4256489

[B36] Luo R. Fang L. Jin H. Wang D. An K. Xu N. (2014). Label-free quantitative phosphoproteomic analysis reveals differentially regulated proteins and pathway in PRRSV-infected pulmonary alveolar macrophages. J. Proteome Res. 13, 1270–1280. 10.1021/pr400852d 24533505

[B37] Ma Q. Zou Y. Lv Y. Song H. Yuan Y. J. (2014). Comparative proteomic analysis of experimental evolution of the *Bacillus cereus–Ketogulonicigenium vulgare* co-culture. PLoS ONE 9:e91789. 10.1371/journal.pone.0091789 24619085 PMC3950281

[B38] Mawuenyega K. G. Forst C. V. Dobos K. M. Belisle J. T. Chen J. Bradbury E. M. (2005). *Mycobacterium tuberculosis* functional network analysis by global subcellular protein profiling. Mol. Biol. Cell 16, 396–404. 10.1091/mbc.E04-04-0329 15525680 PMC539182

[B39] Mesquita F. S. Thomas M. Sachse M. Santos A. J. Figueira R. Holden D. W. (2012). The *Salmonella deubiquitinase* SseL inhibits selective autophagy of cytosolic aggregates. PLoS Pathog. 8:e1002743. 10.1371/journal.ppat.1002743 22719249 PMC3375275

[B40] Miller M. Donat S. Rakette S. Stehle T. Kouwen T. R. Diks S. H. (2010). Staphylococcal PknB as the first prokaryotic representative of the proline-directed kinases. PLoS ONE 5:e9057. 10.1371/journal.pone.0009057 20140229 PMC2816222

[B41] Mujtaba S. Winer B. Y. Jaganathan A. Patel J. Sgobba M. Schuch R. (2013). Anthrax SET protein: a potential virulence determinant that epigenetically represses NF-kappaB activation in infected macrophages. J. Biol. Chem. 288, 23458–23472. 10.1074/jbc.M113.467696 23720780 PMC5395026

[B42] Mukherjee S. Keitany G. Li Y. Wang Y. Ball H. L. Goldsmith E. J. (2006). *Yersinia* YopJ acetylates and inhibits kinase activation by blocking phosphorylation. Science 312, 1211–1214. 10.1126/science.1126867 16728640

[B43] Mukherjee S. Liu X. Arasaki K. McDonough J. Galan J. E. Roy C. R. (2011). Modulation of Rab GTPase function by a protein phosphocholine transferase. Nature 477, 103–106. 10.1038/nature10335 21822290 PMC3206611

[B44] Müller S. Lösche A. Bley T. Scheper T. (1995). A flow cytometric approach for characterization and differentiation of bacteria during microbial processes. Appl. Microbiol. Biotechnol. 43, 93–101. 10.1007/BF00170629

[B45] Neunuebel M. R. Chen Y. Gaspar A. H. Backlund P. S. Jr. Yergey A. Machner M. P. (2011). De-AMPylation of the small GTPase Rab1 by the pathogen *Legionella pneumophila*. Science 333, 453–456. 10.1126/science.1207193 21680813 PMC3209958

[B46] Odendall C. Rolhion N. Forster A. Poh J. Lamont D. J. Liu M. (2012). The Salmonella kinase SteC targets the MAP kinase MEK to regulate the host actin cytoskeleton. Cell Host Microbe 12, 657–668. 10.1016/j.chom.2012.09.011 23159055 PMC3510437

[B47] Okuda J. Toyotome T. Kataoka N. Ohno M. Abe H. Shimura Y. (2005). Shigella effector IpaH9.8 binds to a splicing factor U2AF(35) to modulate host immune responses. Biochem. Biophys. Res. Commun. 333, 531–539. 10.1016/j.bbrc.2005.05.145 15950937

[B48] Parker J. L. Jones A. M. Serazetdinova L. Saalbach G. Bibb M. J. Naldrett M. J. (2010). Analysis of the phosphoproteome of the multicellular bacterium *Streptomyces coelicolor* A3(2) by protein/peptide fractionation, phosphopeptide enrichment and high-accuracy mass spectrometry. Proteomics 10, 2486–2497. 10.1002/pmic.201000090 20432484

[B49] Persson C. Nordfelth R. Andersson K. Forsberg A. Wolf-Watz H. Fallman M. (1999). Localization of the *Yersinia* PTPase to focal complexes is an important virulence mechanism. Mol. Microbiol. 33, 828–838. 10447891 10.1046/j.1365-2958.1999.01529.x

[B50] Price C. T. Al-Quadan T. Santic M. Jones S. C. Abu Kwaik Y. (2010). Exploitation of conserved eukaryotic host cell farnesylation machinery by an F-box effector of *Legionella pneumophila*. J. Exp. Med. 207, 1713–1726. 10.1084/jem.20100771 20660614 PMC2916131

[B51] Prisic S. Dankwa S. Schwartz D. Chou M. F. Locasale J. W. Kang C. M. (2010). Extensive phosphorylation with overlapping specificity by *Mycobacterium tuberculosis* serine/threonine protein kinases. Proc. Natl. Acad. Sci. U.S.A. 107, 7521–7526. 10.1073/pnas.0913482107 20368441 PMC2867705

[B52] Putignani L. Del Chierico F. Petrucca A. Vernocchi P. Dallapiccola B. (2014). The human gut microbiota: a dynamic interplay with the host from birth to senescence settled during childhood. Pediatr. Res. 76, 2–10. 10.1038/pr.2014.49 24732106

[B53] Reiterer V. Grossniklaus L. Tschon T. Kasper C. A. Sorg I. Arrieumerlou C. (2011). *Shigella flexneri* type III secreted effector OspF reveals new crosstalks of proinflammatory signaling pathways during bacterial infection. Cell. Signal. 23, 1188–1196. 10.1016/j.cellsig.2011.03.006 21402152

[B54] Rogers J. B. DuTeau N. M. Reardon K. F. (2000). Use of 16S-rRNA to investigate microbial population dynamics during biodegradation of toluene and phenol by a binary culture. Biotechnol. Bioeng. 70, 436–445. 10.1002/1097-0290(20001120)70:4<436::AID-BIT9>3.0.CO;2-6 11005926

[B55] Royan S. V. Jones R. M. Koutsouris A. Roxas J. L. Falzari K. Weflen A. W. (2010). Enteropathogenic *E. coli* non-LEE encoded effectors NleH1 and NleH2 attenuate NF-kappaB activation. Mol. Microbiol. 78, 1232–1245. 10.1111/j.1365-2958.2010.07400.x 21091507 PMC3325542

[B56] Schirm M. Soo E. C. Aubry A. J. Austin J. Thibault P. Logan S. M. (2003). Structural, genetic and functional characterization of the flagellin glycosylation process in *Helicobacter pylori*. Mol. Microbiol. 48, 1579–1592. 10.1046/j.1365-2958.2003.03527.x 12791140

[B57] Schmidt F. Volker U. (2011). Proteome analysis of host–pathogen interactions: investigation of pathogen responses to the host cell environment. Proteomics 11, 3203–3211. 10.1002/pmic.201100158 21710565

[B58] Schmidt J. K. König B. Reichl U. (2007). Characterization of a three bacteria mixed culture in a chemostat: evaluation and application of a quantitative terminal-restriction fragment length polymorphism (T-RFLP) analysis for absolute and species specific cell enumeration. Biotechnol. Bioeng. 96, 738–756. 10.1002/bit.21147 16937400

[B59] Schmutz C. Ahrne E. Kasper C. A. Tschon T. Sorg I. Dreier R. F. (2013). Systems-level overview of host protein phosphorylation during *Shigella flexneri* infection revealed by phosphoproteomics. Mol. Cell Proteomics 12, 2952–2968. 10.1074/mcp.M113.029918 23828894 PMC3790303

[B60] Scholz R. Imami K. Scott N. E. Trimble W. S. Foster L. J. Finlay B. B. (2015). Novel host proteins and signaling pathways in enteropathogenic *E. coli* pathogenesis identified by global phosphoproteome analysis. Mol. Cell Proteomics 14, 1927–1945. 10.1074/mcp.M114.046847 25944883 PMC4587332

[B61] Seyedarabi A. Sullivan J. A. Sasakawa C. Pickersgill R. W. (2010). A disulfide driven domain swap switches off the activity of Shigella IpaH9.8 E3 ligase. FEBS Lett. 584, 4163–4168. 10.1016/j.febslet.2010.09.006 20831869

[B62] Shui W. Gilmore S. A. Sheu L. Liu J. Keasling J. D. Bertozzi C. R. (2009). Quantitative proteomic profiling of host–pathogen interactions: the macrophage response to *Mycobacterium tuberculosis* lipids. J. Proteome Res. 8, 282–289. 10.1021/pr800422e 19053526 PMC2655317

[B63] Sun F. Ding Y. Ji Q. Liang Z. Deng X. Wong C. C. (2012). Protein cysteine phosphorylation of SarA/MgrA family transcriptional regulators mediates bacterial virulence and antibiotic resistance. Proc. Natl. Acad. Sci. U.S.A. 109, 15461–15466. 10.1073/pnas.1205952109 22927394 PMC3458358

[B64] Tan Y. Arnold R. J. Luo Z. Q. (2011). *Legionella pneumophila* regulates the small GTPase Rab1 activity by reversible phosphorylcholination. Proc. Natl. Acad. Sci. U.S.A. 108, 21212–21217. 10.1073/pnas.1114023109 22158903 PMC3248503

[B65] Thao S. Chen C. S. Zhu H. Escalante-Semerena J. C. (2010). Nepsilon-lysine acetylation of a bacterial transcription factor inhibits Its DNA-binding activity. PLoS ONE 5:e15123. 10.1371/journal.pone.0015123 21217812 PMC3013089

[B66] Tijhuis L. Rekswinkel E. Vanloosdrecht M. C. M. Heijnen J. J. (1994). Dynamics of population and biofilm structure in the biofilm airlift suspension reactor for carbon and nitrogen removal. Water Sci. Technol. 29, 377.

[B67] Tracz D. M. McCorrister S. J. Chong P. M. Lee D. M. Corbett C. R. Westmacott G. R. (2013). A simple shotgun proteomics method for rapid bacterial identification. J. Microbiol. Methods 94, 54–57. 10.1016/j.mimet.2013.04.008 23631909

[B68] Tseng T. T. Tyler B. M. Setubal J. C. (2009). Protein secretion systems in bacterial-host associations, and their description in the Gene Ontology. BMC Microbiol. 9 Suppl. 1), S2. 10.1186/1471-2180-9-S1-S2 19278550 PMC2654662

[B69] Twine S. M. Mykytczuk N. C. Petit M. D. Shen H. Sjostedt A. Wayne Conlan J. (2006). *In vivo* proteomic analysis of the intracellular bacterial pathogen, *Francisella tularensis*, isolated from mouse spleen. Biochem. Biophys. Res. Commun. 345, 1621–1633. 10.1016/j.bbrc.2006.05.070 16730660

[B70] Vilcheze C. Molle V. Carrere-Kremer S. Leiba J. Mourey L. Shenai S. (2014). Phosphorylation of KasB regulates virulence and acid-fastness in *Mycobacterium tuberculosis*. PLoS Pathog. 10:e1004115. 10.1371/journal.ppat.1004115 24809459 PMC4014462

[B71] Voisin S. Watson D. C. Tessier L. Ding W. Foote S. Bhatia S. (2007). The cytoplasmic phosphoproteome of the Gram-negative bacterium *Campylobacter jejuni*: evidence for modification by unidentified protein kinases. Proteomics 7, 4338–4348. 10.1002/pmic.200700483 17973292

[B72] Whitmore S. E. Lamont R. J. (2012). Tyrosine phosphorylation and bacterial virulence. Int. J. Oral. Sci. 4, 1–6. 10.1038/ijos.2012.6 22388693 PMC3412661

[B73] Wiley D. J. Nordfeldth R. Rosenzweig J. DaFonseca C. J. Gustin R. Wolf-Watz H. (2006). The Ser/Thr kinase activity of the *Yersinia protein* kinase A (YpkA) is necessary for full virulence in the mouse, mollifying phagocytes, and disrupting the eukaryotic cytoskeleton. Microb. Pathog. 40, 234–243. 10.1016/j.micpath.2006.02.001 16626927

[B74] Wilmes P. Bond P. L. (2004). The application of two-dimensional polyacrylamide gel electrophoresis and downstream analyses to a mixed community of prokaryotic microorganisms. Environ. Microbiol. 6, 911–920. 10.1111/j.1462-2920.2004.00687.x 15305916

[B75] Wilmes P. Bond P. L. (2009). Microbial community proteomics: elucidating the catalysts and metabolic mechanisms that drive the Earth’s biogeochemical cycles. Curr. Opin. Microbiol. 12, 310–317. 10.1016/j.mib.2009.03.004 19414280

[B76] Xie L. Liu W. Li Q. Chen S. Xu M. Huang Q. (2015). First succinyl-proteome profiling of extensively drug-resistant *Mycobacterium tuberculosis* revealed involvement of succinylation in cellular physiology. J. Proteome Res. 14, 107–119. 10.1021/pr500859a 25363132

[B77] Yang Y. Hu M. Yu K. Zeng X. Liu X. (2015). Mass spectrometry-based proteomic approaches to study pathogenic bacteria-host interactions. Protein Cell 6, 265–274. 10.1007/s13238-015-0136-6 25722051 PMC4383758

[B78] Yarbrough M. L. Li Y. Kinch L. N. Grishin N. V. Ball H. L. Orth K. (2009). AMPylation of Rho GTPases by Vibrio VopS disrupts effector binding and downstream signaling. Science 323, 269–272. 10.1126/science.1166382 19039103

[B79] Zhang C. G. Chromy B. A. McCutchen-Maloney S. L. (2005). Host–pathogen interactions: a proteomic view. Expert. Rev. Proteomics 2, 187–202. 10.1586/14789450.2.2.187 15892564

[B80] Zhang H. Ge Y. (2011). Comprehensive analysis of protein modifications by top-down mass spectrometry. Circ. Cardiovasc Genet. 4, 711. 10.1161/CIRCGENETICS.110.957829 22187450 PMC3320739

